# *Staphylococcus aureus* from patients with chronic rhinosinusitis show minimal genetic association between polyp and non-polyp phenotypes

**DOI:** 10.1186/s12901-018-0064-1

**Published:** 2018-10-16

**Authors:** Jake Jervis Bardy, Derek S Sarovich, Erin P Price, Eike Steinig, Steven Tong, Amanda Drilling, Judy Ou, Sarah Vreugde, Peter-John Wormald, Alkis J Psaltis

**Affiliations:** 1 Department of Otolaryngology Head and Neck Surgery, The Queen Elizabeth Hospital and the University of Adelaide, Woodville South, South Australia SA 5011 Australia; 2 Global and Tropical Health Division, Menzies School of Health Research, Charles Darwin University, Darwin, NT Australia; 3 Present address: Faculty of Science, Health, Education and Engineering, University of the Sunshine Coast, QLD, Sippy Downs, Australia; 4 Present address: Australian Institute of Tropical Health and Medicine, James Cook University, QLD, Townsville, Australia

**Keywords:** Chronic rhinosinusitis, Staphylococcus aureus, Genome-wide association study, Microbial genomics, Whole genome sequencing

## Abstract

**Background:**

*Staphylococcus aureus* has a high prevalence in chronic rhinosinusitis (CRS) patients and is suggested to play a more etiopathogenic role in CRS patients with nasal polyps (CRSwNP), a severe form of the CRS spectrum with poorer surgical outcomes. We performed a microbial genome-wide association study (mGWAS) to investigate whether *S. aureus* isolates from CRS patients have particular genetic markers associated with CRS with nasal polyps (CRSwNP) or CRS without nasal polyps (CRSsNP).

**Methods:**

Whole genome sequencing was performed on *S. aureus* isolates collected from 28 CRSsNP and 30 CRSwNP patients. A mGWAS approach was employed using large-scale comparative genomics to identify genetic variation within our dataset.

**Results:**

Considerable genetic variation was observed, with > 90,000 single nucleotide polymorphisms (SNPs) sites identified. There was little correlation with CRS subtype based on SNPs and Insertion/Delection (Indels). One indel was found to significantly correlate with CRSwNP and occurred in the promoter region of a bacitracin transport system ATP-binding protein. Additionally, two variants of the highly variable superantigen-like (SSL) proteins were found to significantly correlate with each CRS phenotype. No significant association with other virulence or antibiotic resistance genes were observed, consistent with previous studies.

**Conclusion:**

To our knowledge this study is the first to use mGWAS to investigate the contribution of microbial genetic variation to CRS presentations. Utilising the most comprehensive genome-wide analysis methods available, our results suggest that CRS phenotype may be influenced by genetic factors other than specific virulence mechanisms within the *S. aureus* genome.

**Electronic supplementary material:**

The online version of this article (10.1186/s12901-018-0064-1) contains supplementary material, which is available to authorized users.

## Background

The prevalence of chronic rhinosinusitis (CRS) has been reported in the order of 10% in large epidemiological studies [1], with a financial burden in excess of USD$20 billion in the United States annually [2]. CRS is a clinical diagnosis defined by a constellation of symptoms lasting > 12 weeks that include nasal blockage, obstruction, congestion or discharge, facial pain or pressure, and reduction or loss of smell [3]. CRS is phenotypically subclassified into CRS with nasal polyps (CRSwNP) and CRS without nasal polyps (CRSsNP). CRSsNP involves fibrosis and basement membrane thickening, whereas CRSwNP is characterized by an edematous stroma, the formation of pseudocysts, and inflammatory cell infiltrates [4].

Despite recent advances in our understanding of CRS, the etiopathogenesis of this condition remains largely unknown [5]. Theories of CRS pathogenesis can be broadly categorized into environmental and host related factors [6]. The immune barrier hypothesis is the most detailed and inclusive mechanism described in the literature. This theory proposes that a defective host mucosal barrier and the innate immune response predispose CRS patients to mucosal inflammation when colonized by commensal bacteria that would otherwise not cause disease [7].

Central to the environmental component of the immune barrier hypothesis is the role of microbes, in particular the presence of bacterial superantigens, biofilms, intracellular bacteria and dysbiosis of polymicrobial communities in patients with CRS. Common to many of these hypotheses is *S. aureus*. *S. aureus* is an important pathogen with a high prevalence in CRS patients [8] and has been implicated in the pathogenesis of CRS through formation of superantigens [9], biofilms [10] and capacity to survive in mucosal cells [11]. *S. aureus* arguably plays a more etiopathogenic role in patients with CRSwNP, with most culture-based studies finding a higher prevalence of *S. aureus* in patients with polyposis [12]. Additionally, intracellular *S. aureus* and superantigen production have been shown to be more prevalent in CRSwNP patients. The role of *S. aureus* in promoting polyposis is of clinical importance as CRSwNP is generally considered a more severe form of CRS with poorer surgical outcomes.

Based on these prior studies, we hypothesized an underlying differential virulence in *S. aureus* isolated from CRSwNP patients compared to CRSsNP patients and that this variation would have a genetic basis. To test our hypothesis, we first investigated the distribution of well-characterized virulence and antibiotic resistance mechanisms to determine whether certain loci were significantly different between these two cohorts. We then performed a comprehensive microbial genome-wide association study (mGWAS) using both the core and pan-genome of 58 *S. aureus* isolates from CRS patients with and without nasal polyps to determine whether other significant genetic signatures could be identified.

## Methods

### Human ethics and patient inclusion criteria

The study was performed between July 2011 and August 2015 and was approved (HREC/13/TQEHLMH/277) by the human ethics committee at the Queen Elizabeth Hospital (Woodville, South Australia, Australia). Inclusion criteria included patients with both a diagnosis of CRS recalcitrant to appropriate medical therapy and a positive sinus culture for *S. aureus.* Details of patient age, sex, history of prior surgery, type of surgery and diagnosis is included in Additional file 1: Table S1.

### Specimen collection

Mucosal swabs and tissue from patients who underwent surgery during the study period were collected. Culture swabs were also taken in the postoperative period under endoscopic guidance from areas of purulence. Caution was exerted to avoid vestibular contamination by careful retraction of the alar cartilage and the use of guarded culture swabs. Specimens were referred to Adelaide Pathology Partners (APP, Mile End, South Australia, Australia) and processed for bacteriological culture.

### Culture and DNA extraction

Bacteria were isolated on Columbia blood agar, Columbia CNA agar, or cysteine lactose electrolyte deficient agar (Oxoid, Thebarton, SA, Australia). Colonies with *S. aureus* morphology were confirmed to be *S. aureus* using latex agglutination testing [13]. Isolates were subcultured into bovine cerebrospinal fluid broth (Thermo Fisher Scientific, Scoresby, Australia), incubated aerobically overnight and stored in 50% glycerol at − 80 °C. DNA was extracted from the isolates as previously described [14].

### Sequencing, genomics and biostatistical analysis

Genomic data were generated from paired-end Illumina reads using the HiSeq 2000 platform (Illumina, Inc., San Diego, CA, USA). Sequencing was performed at the Australian Genome Research Facility (St Lucia, QLD, Australia) to an average depth of ~ 150×. In silico multilocus sequence typing (MLST) was performed on all genome-sequenced isolates to confirm sequence type (ST) assignments using SRST2v0.1.8 [15], and all strains were confirmed to be *S. aureus* according to a positive *nucA* [16] result upon BLAST analysis. Draft assemblies of the *S. aureus* genomes were constructed using an optimized assembly pipeline of Velvet [17], PAGIT [18], SSPACE [19] and Gapfiller [20], which have been wrapped in MGAP [21, 22]. Draft assemblies were annotated using Prokka v1.11 [23]. Annotated draft assemblies were processed with Roary v3.5.9 [24] to create a pan-genome of all isolates included in the study.

The distribution of variably present virulence and antibiotic resistance loci was assessed to determine if any significant differences existed between the CRSwNP and CRSsNP cohorts. Virulence genes were obtained from the Virulence Factors of Pathogenic Bacteria database (VFDB) [25], which contained 1178 unique sequences at the time of analysis. Antibiotic resistance genes were obtained from the Antibiotic Resistance Gene ANNOTation database (ARG-ANNOT) [26], which contained 1683 unique sequences at the time of analysis. Genes in the VFDB and ARG-ANNOT databases were converted to amino acid sequences using EMBOSS TRANSEQ [27]. The assembled annotated strains were aligned against the converted databases using tBLASTn and normalized using the large-scale BLAST score ratio (LS-BSR) [28]. A BSR value  =  0.8 approximates 80% peptide identity over 100% of the peptide length. A BSR value >.97 was used as the alignment threshold for analysing individual genes. A BSR matrix was constructed with a threshold score ranging between 0.85 to 0.97 to assess association with variable gene presence using PLINK v1.9 [29]. Representative sequences with significant correlation (Bonferroni corrected *p* < 0.05) were aligned to the Microbes database of the National Center for Biotechnology Information (NCBI) using tBLASTx to assess function.

For mGWAS, variants were identified by mapping short read data against the draft assembly of a representative isolate within the CRSwNP group (*S. aureus* DRI_4) using a combination of bwa [30], SAMTools [31] and GATK [32], which are wrapped in the SPANDx v3.1 pipeline [33]. Orthologous biallelic SNPs identified by SPANDx were used as input for maximum parsimony phylogenetic reconstruction using PAUP* v4.0a151 [34]. Matrices of orthologous and non-orthologous SNPs and indels (SPANDx), and presence-absence regions across the pan-genome (Roary) were used as input for mGWAS using PLINK. Bonferroni correction for multiple testing was applied to establish significance.

## Results

### Multilocus sequence typing

Among the 58 *S. aureus* isolates collected for this study, 23 sequence types (STs) were identified. The most common ST was ST5 (9 isolates) followed by ST30 (8 isolates) and ST6 (5 isolates). These three STs have been previously observed in Australian *S. aureus* (https://pubmlst.org/saureus/).

### Correlation of virulence genes with disease presentation

We next assessed virulence and antibiotic resistance gene content within our dataset according to the respective VFDB [25] and ARG-ANNOT^(26)^databases to establish whether any characterized genes were associated with disease state. No significant associations were observed with either database using a chi-squared approach and correction for multiple testing. Similarly, prevalence of membrane damaging toxins Hemolysins a, b and d were similar across the study groups, being identified in 80–100% of isolates (Table 1). Of the bi-component toxins, the gamma toxins HlgA, HlgB and Hlg were also identified in most isolates (96–100%), whereas the Leukocidins were far less prevalent. The LukS/F genes (Panton-Valentine Leukocidin) were not identified in any isolates while the LukD/E genes were identified in over half of the isolates. The family of serine proteases SPL A-F, SspA (V8 protease) and ETA (exfoliative toxin A) were seen in varying prevalence in the isolates studied (Table 2). The V8 protease was identified in almost all isolates (92% CRSsNP, 96% CRSwNP) while the SPLs were seen in 42% (SplA CRSsNP) and 76% (SplF CSRwNP). The cysteine protease Staphopain B (*sspB*) was identified in all assembled genomes. The *sak* gene (Staphylokinase) (75% CRSsNP, 93% CRSwNP) was identified in a higher proportion of genomes than the *coa* gene (Staphylocoagulase) (42% CRSsNP, 30% CRSwNP).

**Table 1:** Distribution of Membrane Damaging Toxins in the polyp vs. non-polyp cohorts

Membrane Damaging Toxins	CRSsNP	CRSwNP	CRSsNP (%)	CRSwNP (%)
Hla hemolysin a	27	28	96%	93%
Hlb hemolysin b	28	30	100%	100%
Hld hemolysin d	27	28	96%	93%
HlgB hemolysin gamma	28	30	100%	100%
HlgA hemolysin gamma	27	30	96%	100%
HlgC hemolysin gamma	23	28	82%	93%
LUK D	16	20	57%	67%
LUK F	0	0	0	0
LUK S	0	0	0	0
LUK E	15	23	54%	77%
LUK M	0	0	0	0

**Table 2:** Distribution of toxin enzymes in the polyp vs. non-polyp cohorts

Enzymes	CRSsNP	CRSwNP	CRSsNP (%)	CRSwNP (%)
SplB	15	20	54%	67%
SplD	18	21	64%	70%
SplF	17	23	61%	77%
SplC	19	19	68%	63%
SplE	13	13	46%	43%
SplA	12	17	43%	57%
SspB - Staphopain B	28	30	100%	100%
SspA - V8 protease	26	29	93%	97%
ETA Exfoliative Toxin A	27	29	96%	97%
Staphylokinase	21	28	75%	93%
Staphylocoagulase	12	9	43%	30%

Fourteen of the known superantigen toxin genes (SEA/B/C/G/H/I/K/L/M/O/P/Q/U/TSST) were identified in the *S. aureus* isolates (Table 3). Only three superantigen toxins were identified in > 50% of the isolates (SEG, SEM, SEO). The remaining toxins were identified in < 25% of genomes. Despite marked variation in the prevalence of the various enterotoxins (3% for sek to 54–63% for seg), no significant difference was identified across the two isolate groups. The ICA locus genes (ICA A,B,C,D,R), which are involved in biofilm formation, were observed with a very high prevalence in both groups, with over 90% of isolates carrying the gene (Table 4). The fibronectin binding proteins FnbA/FnbB, thought to be involved in cell wall internalisation, were observed with less prevalence in all groups (16–28%).

**Table 3:** Distribution of superantigens (SAGs) in the polyp vs. non-polyp cohorts

SAGs	CRSsNP	CRSwNP	CRSsNP (%)	CRSwNP (%)
SEA	5	7	17%	23%
SEB	2	4	7%	13%
SEC	4	2	14%	7%
SEG	15	19	54%	63%
SEH	4	1	14.29%	3%
SEI	16	16	58%	53%
SEK	1	1	4%	3%
SEL	4	1	14%	3%
SEM	17	17	61%	57%
SEO	17	16	61%	53%
SEP	2	3	7%	10%
SEQ	2	2	7%	7%
SEU	5	5	18%	17%
TSST	4	7	14%	23%

**Table 4:** Distribution of cell wall-associated toxin proteins in the polyp vs. non-polyp cohorts^a^

Cell wall associated proteins	CRSsNP	CRSwNP	CRSsNP (%)	CRSwNP (%)
FNBa	12	9	43%	30%
FNBb	8	6	28.57%	20%
IcaA	28	30	100.00%	100%
IcaB	25	29	90%	97%
IcaC	27	29	97%	97%
IcaD	27	30	96%	100%
IcaR	28	29	100%	97%

^a^After Bonferroni correction for multiple testing, no toxin genes in Tables 1, 2, 3 and 4 were found to reach a significance threshold and therefore individual *P* values have been omitted

### Correlation of antibiotic resistance genes with disease presentation

**Fig. 1:**
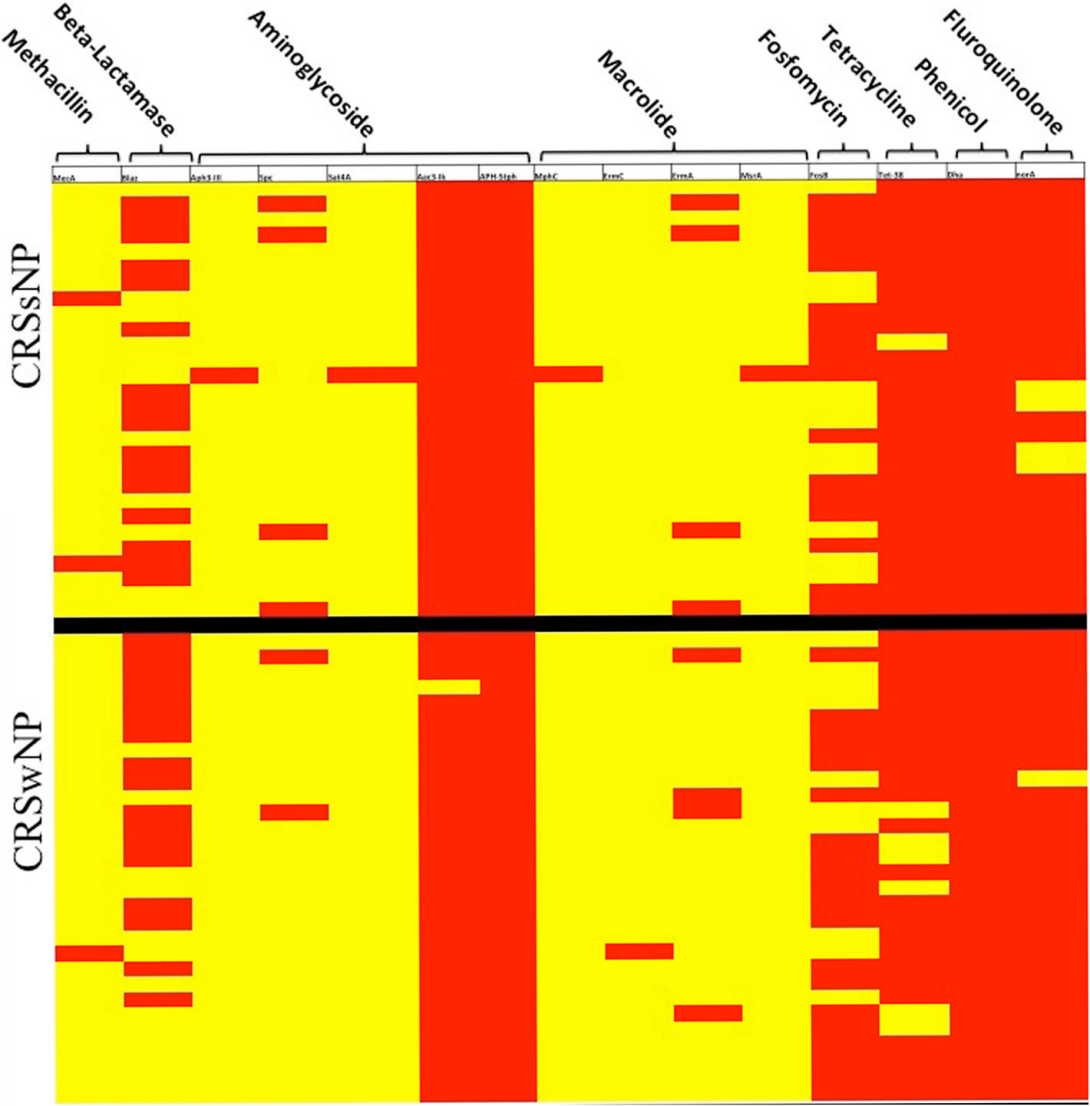
Gene presence-absence matrix of antibiotic resistance loci for the CRSwNP- and CRSsNP-derived *S. aureus* strains. Red, gene presence; yellow, gene absence

The most common antibiotic resistance determinants encoded genes for aminoglycoside resistance (Aac3-lk, Sat4A, APH-Stph, APH-3, Spc) and phenicol (Dha1) resistance, with these determinants found in all isolates (Fig. 1). Tetracycline (Tet-38; 96% CRSsNP, 87% CRSwNP), fluoroquinolone (NorA; 86% CRSsNP, 97% CRSwNP), β-lactamase (BlaZ; 57% CRSsNP, 57% CRSwNP) and fosfomycin (FosB; 61% CRSsNP, 67% CRSwNP) resistance genes were also highly prevalent. As expected, macrolide resistance genes (MphC, ErmC, ErmA, MsrA; 18% CRSsNP, 13% CRSwNP) and the MecA cassette for methicillin resistance (7% CRSsNP, 3% CRSwNP) were rarely identified.

### Correlation of core genome variation with disease presentation

Comparative genomic analysis identified 92,474 SNPs and 2031 indels across the core genome of the 30 CRSwNP-derived and 28 CRSsNP-derived *S. aureus* isolates. These SNPs were used to construct a maximum-parsimony phylogenetic tree. Consistent with the MLST results, no clear phylogenetic association was seen in the CRSwNP or CRSsNP groups. mGWAS also revealed no significant associations using core genome SNPs, with all failing to reach significance level of a Bonferroni corrected *p* < 0.05 (Additional file 2: Table S2a). In contrast, one small indel in the core genome was significantly associated with CRSwNP isolates (Bonferroni corrected *p* = 0.036). This indel occurred in the promoter region of *SAOUHSC_02954* (NCTC8325 annotation), which encodes a bacitracin transport system ATP-binding protein (Additional file 2: Table S2b). A second indel in the accessory genome also reached significant level; however, this variant was not found to be in a protein-coding or promoter region.

mGWAS was also carried out on the pan-genome of the CRSwNP and CRSsNP isolates, comprising 5978 genes. Of these, 1783 genes were contained in the core genome while the remaining 4015 were variable (Additional file 2: Table S2c). Assessment of the pan-genome content for correlation with disease presentation showed two genes reaching a significance level (Bonferroni corrected *p* < 0.05). The first was superantigen-like protein 5 (Bonferroni corrected *p* = 0.017) and the second was superantigen-like protein 14 (Bonferroni corrected *p* = 0.023).

## Discussion

To our knowledge, this study is the first reported mGWAS investigating the genetics of microbes and CRS phenotypes. We found considerable variation within our *S. aureus* dataset, with > 90,000 SNPs identified from 23 STs and almost 6000 genes comprising the pan-genome. Despite this variation, there were few significant associations observed between the genetic variation and CRS phenotypes tested. One indel in the core genome of the isolates was found to reach corrected significant thresholds, while two highly variable genes were found to reach corrected significance in the accessory genome. Assessment of the pan-genome content for correlation with disease presentation showed two superantigen like toxin genes (SSL genes) that reached significance thresholds. SSL5 was identified in higher prevalence in the CRSsNP cohort, while SSL14 was more prevalent in CRSwNP cohort. The SSL genes have been given little attention in studies of S. aureus virulence, with a limited number of reports in the general literature and no reports in rhinology research. These proteins are thought to impede neutrophil migration and complement stimulation, as opposed to T-cell activation that is commonly associated with traditional superantigens [35, 36]. The role of SSLs in the pathogenesis of CRS is nevertheless unknown without functional verification.

Testing for the genetic basis of disease was first pioneered in human genetics using genetic linkage methods. This approach was only appropriate in specific monogenic disorders (such as cystic fibrosis) where single gene mutations caused the relevant disorder, with disease causing alleles tracked though generations due to recombination events secondary to sexual reproduction [37]. In complex trait genetics where various genes and environmental factors contribute to a disease phenotype, it was not possible to use traditional approaches relying on the principles of Mendelian inheritance. Instead, there has been a revolution in the study of complex trait genetics in the form of GWAS, where thousands of SNPs can be tested simultaneously for association with disease. In human genetics, this approach has led to the discovery of loci associated with various human diseases that have been subsequently targeted by novel medical therapies [38]. As GWAS rely on large-scale comparisons (as opposed to linkage-based analysis), it is possible to translate this work into the field of microbial genomics [39].

The use of microbial GWAS (mGWAS) in microbial genomics is relatively new, with the first successful mGWAS published in 2013 [40], and other studies published since then [41, 42]. This approach has also recently been used to study *S. aureus*, with Read and colleagues [39] identifying 121 genetic loci significantly associated with changes in the toxicity of individual MRSA isolates of the same sequence type (ST239) [43]. In the field of rhinology however, all existing studies investigating the genetics of *S. aureus* and CRS phenotypes have used a “bottom-up” approach, surveying known virulence or resistance genes across CRSwNP, CRSsNP and control groups [44, 45]. Thunberg et al [45] used a DNA microarray to detect 170 distinct virulence and antibiotic resistance genes in 18 CRS patients and 16 controls, finding no significant difference in gene prevalence. Heymans et al [44] used a similar approach but with PCR to screen for 22 exotoxin genes (enterotoxins and exfoliative toxins A and B) in 55 CRSwNP, 16 CRSsNP and 22 control patients; they also found no significant difference in prevalence. Investigating genetic association across only a small gene panel risks missing truly significant associations across the genome, and it can lead to erroneous conclusions due to linkage disequilibrium, particularly for organisms exhibiting relatively low recombination rates like *S. aureus*. In addition, gene presence/absence is only one form of genetic variation, with SNPs and indels being potentially important in a given phenotype. The use of mGWAS is therefore the only feasible approach to determining whether there is a precise genetic basis in the *S. aureus* genome that accounts for different CRS phenotypes [39].

The possibility that our study has indeed identified *S. aureus* point mutations or indels that causes polyp or non-polyp disease is unlikely, due to the low level of correlation found. Read and colleagues recently identified 121 significant loci associated with toxicity in an mGWAS of 90 methicillin-resistant *S. aureus* (MRSA) isolates. The authors selected 13 of the significant polymorphisms that were thought to affect toxicity, finding that only four of these regions affected T-cell survival in vitro when verified using transposon mutagenesis. Considering our study has only uncovered four regions of interest (two indels and two SSL genes), it is highly likely these associations will be false positives due to the large number of variants tested. Future work involving increased sample size may be able to detect weaker associations not able to be elucidated in our study.

We further targeted known virulence and resistance genes in silico, to establish the prevalence of previously described genes and to compare this cohort with previous studies of *S. aureus* in CRS. Similar to the previous literature, there were no significant differences in the prevalence of the virulence genes tested across CRSwNP and CRSsNP isolates [44, 45]. There was a high prevalence of some membrane damaging toxins (Hemolysins a, b, d and HlgA, HlgB and Hlg), while the leukocidins were far less prevalent, with the LukS/F genes (Panton-Valentine Leukocidin (PVL)) not identified in any isolates. The protease genes were seen in varying prevalence in the isolates studied. Fourteen of the known superantigen (SAG) toxin genes (Sea/b/c/g/h/I/k/l/m/o/p/q/u/TSST) were identified in the isolates with varying prevalence. SEG, SEM and SEO (of the ECG cluster) were the most commonly identified. The SAG’s have been the most studied virulence genes in the CRS literature. We found similar prevalence of the most common SAG’s reported in these studies confirming that there is variation between the SAG’s and that the ECG cluster, is the most ubiquitous. [44–46] In relation to cell wall associated proteins, the ICA locus genes (ICA A,B,C,D,R), involved in biofilm formation were observed with a very high prevelance, of over 90% of isolates carrying the gene. The fibronectin binding proteins FnbA/FnbB, thought to be involved in cell wall internalization, were observed with less prevalence in all groups (from 16 to 28%). In relation to antibiotic resistance genes, screening results were as expected, with methicillin and macrolide resistance genes rare and other antibiotic resistance genes far more common (eg. the aminoglycoside, phenicol, tetracycline, fluroquinolone, β-lactamase and fosfomycin resistance genes).

There were a number of limitations inherent in this study. Firstly, a single colony of *S. aureus* was selected for sequencing from each patient, raising the possibility that this may not be the disease-associated *S. aureus* strain but rather a non-invasive commensal strain. We did not sequence control *S. aureus* isolates patients in the current study, as our hypothesis was whether there exists an association between *S. aureus* virulence and CRS phenotypes. We acknowledge that the number of samples used in this study were modest and that a greater number of isolates would be required to identify a smaller effect size. Further mGWAS using all CRS strains (CRSwNP and CRSwoNP) as a disease group and non-CRS strains as a control group may lead to the identification of genetic loci in *S. aureus* that contribute to the pathogenesis of CRS, and should be the focus of future mGWAS. We have not verified the significant associations identified in this study using functional characterisation, so inferring gene expression remains speculative. In light of our findings, it is likely that the spectrum of disease in CRS may be more related to host genetics and environmental factors than a response to specific *S. aureus* virulence mechanisms.

## Conclusions

To our knowledge this is the first reported mGWAS investigating the effect of microbes on CRS phenotypes. We found considerable variation among the *S. aureus* isolates from 58 patients with CRS; however, there were minimal significant associations observed between the CRS phenotypes tested. Utilising the most comprehensive genome-wide analysis methods available, our results suggest that CRS phenotypes may be influenced by factors other than specific virulence mechanisms within the *S. aureus* genome.

## Additional files

Additional file 1: **Table S1.** Patient characteristics. (XLSX 13 kb)

Additional file 2: **Table S2a**. Top SNP hits using mGWAS. **Table S2b**. Top indel hits using mGWAS. **Table S2c**. Top pan-genome hits using mGWAS. (XLSX 29 kb)

## Abbreviations

ARG-ANNOT: Antibiotic Resistance Gene ANNOTation database (ARG-ANNOT)
BSR: BLAST score ratio
CRS: Chronic rhinosinusitis (CRS)
CRSsNP: CRS without nasal polyps
CRSwNP: CRS with nasal polyps
Indel: Insertion/Deletion
MGWAS: Microbial genome-wide association study (mGWAS)
NCBI: National Center for Biotechnology Information
SNP: Dingle nucleotide polymorphism
SSL: Superantigen-like (SSL) proteins
ST: Sequence type
VFDB: Virulence Factors of Pathogenic Bacteria database (VFDB)
